# Expression and Prognostic Significance of PDIA3 in Cervical Cancer

**DOI:** 10.1155/2022/4382645

**Published:** 2022-11-11

**Authors:** Jing Zhang, Hui Li, Huling Li, Dandan Lin, Xiaoyan Wang, Kai Wang

**Affiliations:** ^1^School of Public Health, Xinjiang Medical University, Urumqi 830017, China; ^2^Central Laboratory, Xinjiang Medical University, Urumqi 830011, China; ^3^Department of Medical Engineering and Technology, Xinjiang Medical University, Urumqi 830017, China

## Abstract

To investigate the expression of protein disulfide isomerase A3 (PDIA3/ERP57) in cervical cancer and its clinical prognostic significance as well as its function and possible action mechanism in the progression of cervical cancer. Based on TIMER2.0 database, the human protein map (Human Protein Atlas) was used to determine the expression level of PDIA3 protein for the analysis of PDIA3 expression in 39 The Cancer Genome Atlas (TCGA) tumors. The PDIA3 expression in cervical cancer tissues in the TCGA and Genotype-Tissue Expression databases was further verified based on the GEPIA2 database to analyze the relationship between the PDIA3 expression and the pathological stage of cervical cancer patients. Immunohistochemistry was used to detect the PDIA3 expression in cervical cancer tissue microarray, including 111 cancer tissue samples and 24 adjacent cancer tissue samples, and the relationship between PDIA3 protein expression and clinical characteristics of patients with cervical cancer was analyzed. The Kaplan–Meier method and log-rank test were used for survival analysis. Based on the cBioPortal database, the Spearman's and Pearson's methods were used to analyze the correlation between PDIA3 expression and DNA methylation. The correlation between PDIA3 expression and the infiltration levels of each immune cell in cervical cancer was evaluated. The STRING was used to construct protein interaction network. Based on LinkedOmics database, the Spearman's method was used to analyze the co-expressed genes of PDIA3 in TCGA cervical cancer. The gene ontology functional enrichment analysis was performed on Top 50 differentially co-expressed genes based on DAVID database. The PDIA3 expression in cervical cancer tissues was significantly higher than that in normal tissues, which (*F* = 2.74, PR (>*F*) = 0.0436) was significantly increased with the progression of tumor stage, and PDIA3 showed strong immunoreactivity in cervical cancer tissues. In cervical cancer patients, overall survival (*P* = 0.014), disease-specific survival (*P* = 0.013), disease-free interval (*P* = 0.023), and progression-free interval (*P* = 0.001) in those with high expression of PDIA3 were significantly lower than those with low expression, suggesting that high expression of PDIA3 was associated with poor prognosis. In cervical cancer, high expression of PDIA3 was associated with DNA methylation and negatively correlated with B cell memory (*r* = −0.132, *P* = 0.021), T cell regulatory (*r* = −0.127, *P* = 0.026), monocytes (*r* = −0.204, *P* = 0), and macrophages M2 (*r* = −0.142, *P* = 0.013), whereas positively correlated with levels of NK cell activated (*r* = 0.162, *P* = 0.005) and mast cells activated (*r* = 0.119, *P* = 0.037). The genes positively correlated with PDIA3 expression included HSPA5 and PPIB, which were mainly enriched in biological processes, such as endoplasmic reticulum (ER) protein folding and ER stress response. PDIA3 can be used as a marker of poor prognosis of cervical cancer. The expression level of PDIA3 is closely related to the survival and prognosis of cervical cancer patients, DNA methylation, and immune cell infiltration.

## 1. Introduction

Cervical cancer is the fourth most commonly diagnosed cancer and the fourth leading cause of death among women due to cancer. In 2020, it was estimated that there were 604,000 new cases worldwide, and 342,000 patients died from cervical cancer, of which 108,000 new cases of cervical cancer and about 50,000 related deaths occurred in China every year. Most cervical cancer patients can be prevented from routine screening, such as human papillomavirus (HPV) vaccination, early screening, surgical treatment, chemoradiotherapy, and targeted therapy and treatment of precancerous lesions [1–4]. However, cervical cancer continues to be a serious threat to women's health and lives due to inadequate screening programmers in many parts of the world. In recent years, with the gradual acceleration of the life pace in China, the mortality and morbidity of female cervical cancer patients show increasing and younger trends [5]. Like other malignant tumors, cervical cancer's specific gene expression regulates the biological characteristics of cervical cancer cells and promotes the growth, differentiation, invasion, and metastasis of cervical cancer cells. Although a variety of pathological mechanisms related to the progression of cervical cancer have been proposed, further research is still needed to reveal the occurrence and development process of cervical cancer.

Protein disulfide isomerase A3 (PDIA3/ERP57) is a chaperone protein that is primarily localized to the endoplasmic reticulum (ER). Under normal circumstances, the major part of PDIA3 is associated with the ER and forms complex with calreticulin and calnexin to participate in the correct folding and quality control of new synthetic glycoproteins secreted or localized to cell membranes, which is not only capable of protein modification and folding but also capable of exerting the effects of group thinning and oxidation reduction [6]. PDIA3 is expressed in various types of human cancers, including ovarian cancer, breast cancer, uterine cancer, lung cancer, gastric cancer, and hepatocellular carcinoma [7], and its expression is related to the prognosis and survival of various cancers [8, 9]. For example, in patients with diffuse glioma, clear cell renal cell carcinoma, and hepatocellular carcinoma, high expression of PDIA3 is associated with poor survival outcomes [10]. PDIA3 can be highly expressed in immune cells, which is involved in a variety of cytological functions and participates in multiple biological processes, including assembly of MCH-I, regulation of STAT3 pathway, and mediation of apoptosis mechanisms. This study aims to investigate the expression of PDIA3/ERP57 in cervical cancer patients and clinical significance, as well as its impact on the prognosis of patients.

## 2. Materials and Methods

### 2.1. Data Sources

Based on the tumor immune database TIMER2.0 (http://timer.cistrome.org/), Gene_DE under Exploration model was used to evaluate the differential expression between all tumor tissue and adjacent normal tissue of PDIA3 in the public database cancer genome atlas database (The Cancer Genome Atlas [TCGA], https://portal.gdc.cancer.gov/), and the Human Protein Atlas (HPA; https://www.proteinatlas.org/) database was used to determine the protein expression levels of PDIA3. The “Pathological Stage Plot” module based on the GEPIA2 (http://gepia2.cancer-pku.cn/#analysis) database was used to further verify the expression of PDIA3 in the TCGA and the Genotype-Tissue Expression (GTEx) and the relationship with cervical cancer tumor stages. The screening conditions were set as follows: |log2FC| cutoff: 1, *P*-value cutoff: 0.05, cervical squamous and adenocarcinoma (CESC), Match TCGA normal, and GTEx data, including 306 cervical cancer tissue samples and 13 normal tissue samples. There were totally 309 cases of gene expression data of each sample of cervical cancer patients downloaded from the TCGA database, including 306 cases of cervical cancer tissue and 3 cases of normal cervical tissue. The gene expression data were subjected to log2 (TPM + 0.001) standardization, and the gencode.v22.annotation.gtf.gz gene annotation file was downloaded from the GENCODE (https://www.gencodegenes.org/) database. The survival data and clinical phenotypic data of cervical cancer patients were extracted, excluding samples without complete survival information and survival time less than 30 days. Finally, 276 samples of cervical cancer patients were obtained. Four survival indicators including overall survival (OS), disease-specific survival (DSS), disease-free interval (DFI), and progression-free interval (PFI) were selected to investigate the relationship between PDIA3 expression and the prognosis of cervical cancer patients. The immune cell infiltration degree data were downloaded from National Cancer Institute (NCI)-based cancer genome data sharing genomic data commons(GDC) database (https://gdc.cancer.gov/about-data/publications/panimmun), and R software packages “ggplot2,” “ggpubr,” and “ggExtra” were used to evaluate the correlation between the expression level of PDIA3 in cervical cancer and the infiltration level of each immune cell (*P* < 0.05). A PPI network for PDIA3 was built based on a STRING (http://string-db.org) database.

### 2.2. Main Materials

The main materials include antigen repair solution EnVision™ FLEX+, mouse, and high pH (link; Agilent Technologies, Glostrup, Denmark); anti body diluent with background reducing components (Agilent Technologies); endogenous biotin blocking kit (Maixin Biotechnology, Fuzhou, China); ultrasensitive SP kit (Maixin Biotechnology); rat IgG-immuno-histochemical kit (Boster Biological Technology, Wuhan, China); PDIA3 primary antibody (Boster Biological Technology); and cervical cancer tissue microarray HUteS136Su01 (Shanghai Outdo Biotech Company, Shanghai, China).

### 2.3. Method

#### 2.3.1. Expression of PDIA3 in Cervical Cancer and Pathological Staging Analysis

Based on TIMER2.0 database, Gene_DE under Exploration module was used to retrieve the differential expression of PDIA3 in all tumor tissues and adjacent normal tissues in TCGA database, and Wilcoxon test was performed for these tumor types. If *P* < 0.05, it was considered to be differentially expressed between tumor tissues and normal tissues, and the distribution of gene expression level was shown by box diagram. In this study, the expression of PDIA3 in cervical cancer tissues in the TCGA and GTEx databases was further verified based on the GEPIA2 database, and the relationship between the expression of PDIA3 and the pathological stages of cervical cancer patients was analyzed.

#### 2.3.2. Analysis of the Relationship between the Expression of PDIA3 and the Prognosis of Cervical Cancer Patients

Survival and clinical phenotyping data were extracted from each sample of cervical cancer patients downloaded from the public database cancer genome atlas, excluding samples without complete survival information and with survival time less than 30 days. Finally, 276 samples of cervical cancer patients were obtained [11]. Four survival indicators including OS, DSS, DFI, and PFI were selected to investigate the relationship between PDIA3 expression and the prognosis of cervical cancer patients. The Kaplan–Meier method and log-rank test were used for survival analysis (*P* < 0.05), and R software packages “survival” and “survminer” were used to draw survival curves and analyze the relationship between the PDIA3 expression and the prognosis of cervical cancer patients.

#### 2.3.3. Study on the Correlation between PDIA3 Expression and DNA Methylation

In this study, HM450 methylation data from the cBioPortal (http://www.cbioportal.org/) database was used to analyze the change in the genomic level of PDIA3 expression in two groups of cervical cancer tissue samples, one group of cervical cancer tissue samples from the TCGA database and the other group from GDAC Firehose source data. There were 607 cervical cancer patients in total, and the screening conditions were as follows: copy number alterations) and mutations (Mut). Spearman's and Pearson's tests in the plot module were used to analyze the correlation between PDIA3 expression and DNA methylation.

#### 2.3.4. Relationship between PDIA3 Expression Level and Tumor Immune Cell Infiltration Level

Based on the infiltration degree data of immune cells downloaded from the cancer genome data sharing GDC database (https://gdc.cancer.gov/about-data/publications/panimmune) of the NCI, the infiltration of non-tumor cells was predicted by analyzing the specific gene expression characteristics of immune cells and stromal cells. The R software packages “ggplot2”, “ggpubr,” and “ggExtra” were used to evaluate the correlation between the expression level of PDIA3 in cervical cancer and the infiltration level of each immune cell, with *P* < 0.05 considered to be statistically significant.

#### 2.3.5. PPI Network Construction and Related Protein Interaction Analysis of PDIA3

In this study, STRING (http://string-db.org) was used to construct the PPI network of PDIA3, with the overall score > 0.4 considered to be statistically significant. The GEPIA database “Similar Genes Detection” was used to analyze the correlation between the top 10 genes and PDIA3 expression, and the heat map visualization was used to display the expression of 10 target genes in each tumor.

#### 2.3.6. Differential Co-Expression Gene Screening and Functional Enrichment Analysis of PDIA3 in Cervical Cancer

The LinkedOmics-based database (http://linkedomics.org/login.php) was used to analyze the differentially co-expressed genes of PDIA3 in cervical cancer patients in TCGA database, including 304 cervical cancer patients, and to retrieve the up-regulated and down-regulated genes of PDIA3 in cervical cancer. The screening conditions were as follows: TCGA_CESC, RNAseq, PDIA3, RNAseq, and Spearman's correlation test for data analysis. *P* < 0.05 was to be statistically significant, and the top 50 genes with the closest differentially co-expressed genes were selected to make the heat map. The first 50 differential co-expressed genes most closely related to PDIA3 co-expressed genes screened out based on the DAVID (https://david.ncifcrf.gov/) data-base were subjected to gene ontology (GO) functional enrichment analysis. The enrichKEGG () function in the R software package “clusterProfiler” was used to conduct Kyoto Encyclopedia of Genes and Genomes(KEGG) pathway analysis, and the gene names of the first 50 differentially co-expressed genes were screened most closely related to PDIA3 underwent gene ID conversion, generating a bubble chart [12]. The R software package “pathview” was used to analyze the positional relationship of PDIA3 differentially co-expressed genes in the pathway.

#### 2.3.7. Immunohistochemical Staining

The cervical cancer tissue microarray was placed in an oven at 63°C and waxed for one hour. The 1.5 mm slides were used for immunostaining. After dewaxing, these slides were placed in an antigen repair apparatus, and after repairing, they were placed in distilled water at room temperature and allowed to cool naturally for more than 10 minutes. The primary antibody incubation was washed with phosphate buffered saline(PBS) buffer solution and added with diluted primary antibody working solution, in a 4°C refrigerator overnight. The slide was taken from the secondary antibody incubation refrigerator, rewarmed for 45 minutes at room temperature, and washed with PBS buffer, put into a DAKO immunohistochemistry autostainer. As per the “Autostainer Link 48 User Guide,” the blocking, secondary antibody combination and DAB color rendering procedures ran by selecting the corresponding program. Then, the slices were restained with hematoxylin for 1 minute, immersed in 0.25% hydrochloric acid alcohol (400 ml 70% alcohol + 1 ml concentrated hydrochloric acid) for about 10 seconds, and rinsed with running water for 5 minutes. Slides were dried at room temperature and sealed with neutral resin.

#### 2.3.8. Determination of Results

PDIA3 staining was mainly distributed in the cytoplasm, which was positive in case of the presence of brown-yellow particles in the cytoplasm and negative in the case of the absence of brown particles in the cytoplasm or consistence with background color. Double-blind immunohistochemistry score was performed by two experienced pathologists. First, the scores were recorded according to the staining intensity of positive cells, with 0 for basically no staining, 1 for light brown, 2 for pale brown, and 3 for dark brown. The scores were then conducted according to the percentage of positive cells in the counted cells under the microscope, with 0 for ≤5% 1 for 6–30%, 2 for 31–80%, and 3 for >80%. The final score = the score of staining degree × the score of percentage of positive cells. The final score of 0–2 was defined as low expression, and 3–9 as high expression.

## 3. Results

### 3.1. Differential Expression Analysis of PDIA3 in Pan-Cancer

**Figure 1:**
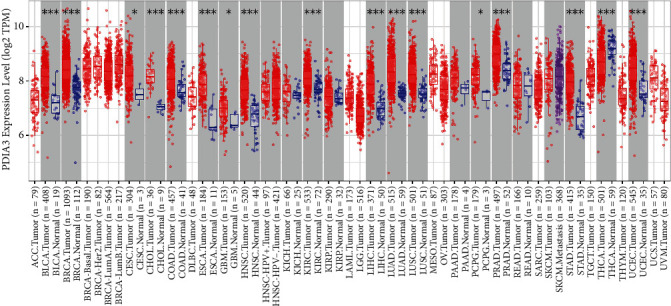
Expression of PDIA3 in various TCGA tumor tissues (∗*P* < 0.05, ∗∗*P* < 0.01, ∗∗∗*P* < 0.001).

**Figure 2:**
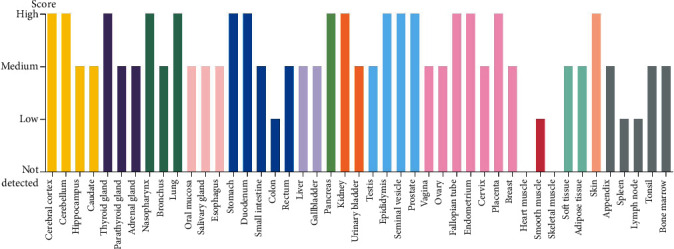
Protein expression levels of PDIA3 in various HPA tumor tissues.

The expression of PDIA3 in 39 TCGA tumors was analyzed based on the TIMER2.0 database. The results showed that PDIA3 was significantly and highly expressed in 16 cancers including bladder urothelial carcinoma, breast cancer, CESC, cholangiocarcinoma (CHOL), colon cancer, esophageal cancer, glioblastoma multiforme, head and neck squamous cell carcinoma, renal clear cell carcinoma, liver hepatocellular carcinoma, lung adenocarcinoma, lung squamous cell carcinoma, pheochromocytoma and paraganglioma, prostate adenocarcinoma, stomach adenocarcinoma, and uterine corpus endometrial carcinoma. In thyroid carcinoma, PDIA3 expression was down-regulated in tumor tissues compared with normal tissues. However, PDIA3 expression levels were not significantly different between kidney chromophobe, kidney renal papillary cell carcinoma, pancreatic adenocarcinoma, and rectum adenocarcinoma cancers and those with only normal tissue samples (Figure 1). In TCGA_CESC, compared with the paracancerous (*n* = 3), the expression level of PDIA3 in the cervical cancer tissue (*n* = 304) was significantly increased (*P* < 0.05 represents a significant difference), and PDIA3 showed strong immunoreactivity in the cervical cancer tissue (Figure 2) [13].

### 3.2. Expression of PDIA3 in Cervical Cancer and Pathological Staging Analysis

**Figure 3:**
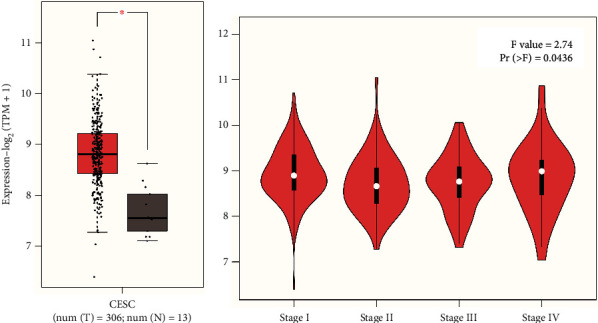
(a) Expression of PDIA3 gene in normal tissue and tumor tissue. (b) Relationship between PDIA3 expression and tumor staging.

Based on the GEPIA2 database, the expression of PDIA3 in 306 cervical cancer cases in the TCGA and GTEx databases was further verified, including 306 cervical cancer tissue samples and 13 normal tissue samples, and the expression levels of PDIA3 in cervical cancer tissues and normal tissues were compared. The results showed that the expression level of PDIA3 in cervical cancer tissue was significantly higher than that in normal tissue (Figure 3(a)), and the difference was statistically significant (*P* < 0.05). Furthermore, the relationship between PDIA3 and cervical cancer tumor staging was analyzed based on the GEPIA2 database, to preliminarily explore the distribution of PDIA3 in cervical cancer tumor staging and draw the violin figure of tumor staging. The results showed that the expression level of PDIA3 (*F* = 2.74, PR (>*F*) = 0.0436) was significantly increased with the progression of tumor staging (Figure 3(b)), and the difference was statistically significant. This indicates that PDIA3 is highly expressed in cervical cancer and can be used as one of the indicators for poor prognosis of late cervical cancer patients.

### 3.3. Expression of PDIA3 in Cancer and Adjacent Cancer Tissues of 111 Cervical Cancer Patients

**Figure 4:**
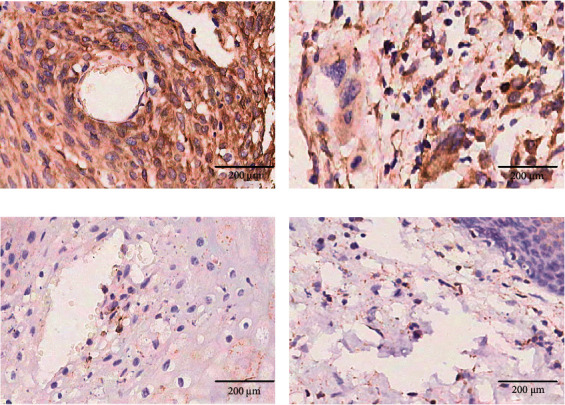
Expression of PDIA3 in cervical cancer and adjacent cancer tissues (IHC 200×). (a) and (b) The positive expression of PDIA3 protein in cervical cancer samples. (c) and (d) The negative expression of PDIA3 protein in normal tissue samples.

Immunohistochemical staining showed that PDIA3 protein was mainly expressed in the cytoplasm. There were 67 PDIA3 protein positive expression cases (60.4%) and 44 negative expression cases (39.6%) in 111 cervical cancer tissues. Among the 24 adjacent cancer tissues, there were 8 (33.3%) PDIA3 protein positive expression cases and 16 (66.7%) negative expression cases. The positive expression rate of PDIA3 in cervical cancer tissues was higher than that in the adjacent cancer tissues (Figures 4(a), 4(b), 4(c), and 4(d)), and the difference was statistically significant (*χ*^2^ = 4.7946, *P* < 0.05). Moreover, the percentages of high expression of PDIA3 protein in cervical cancer tissues were higher than those in the adjacent cancer tissues, and the difference was statistically significant (*P* < 0.05), as shown in Table 1.

### 3.4. Expression Characteristics of PDIA3 in Cervical Cancer Tissue and Its Relationship with Clinicopathological Characteristics

The chi-squared test or Fisher's exact test was used to analyze the correlation between high or low expression of PDIA3 and clinicopathological factors in patients with cervical cancer. As shown in Table 2, the high or low expression of PDIA3 in cervical cancer tissues was not significantly correlated with age, recurrence, T stage, and N stage (*P* > 0.05), but significantly correlated with pathological type (*P* = 0.0004).

### 3.5. Prognostic Value of PDIA3 Expression in Patients with Cervical Cancer

**Figure 5:**
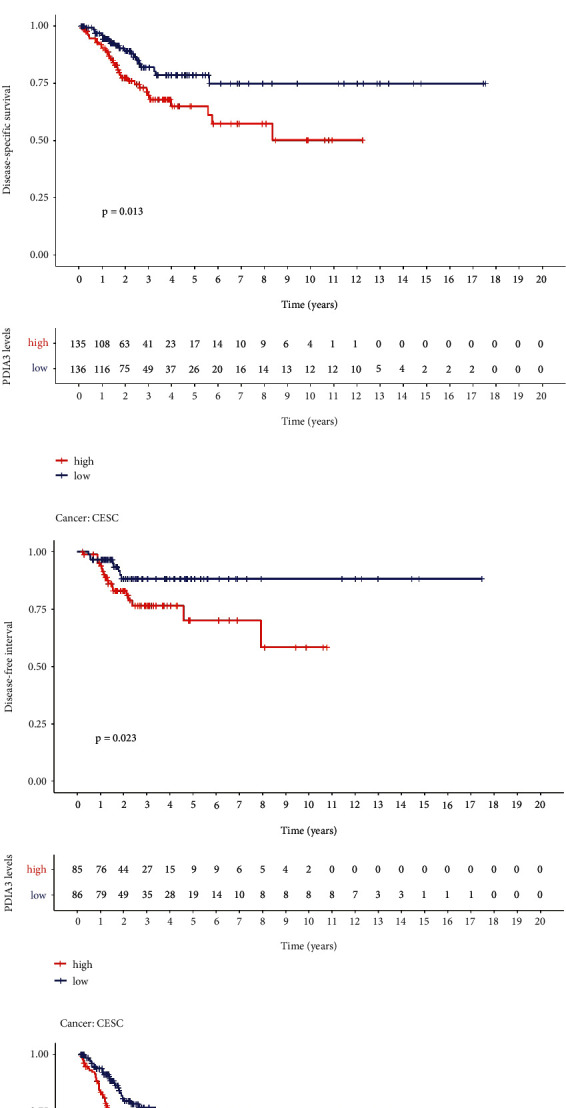
(a) Relationship between PDIA3 expression and overall survival (OS). (b) Relationship between PDIA3 expression and disease-specific survival (DSS). (c) Relationship between PDIA3 expression and disease-free interval (DFI). (d) Relationship between PDIA3 expression and progression-free interval (PFI).

The Kaplan–Meier method and log-rank test were used to analyze the survival of the selected four survival indicators, namely, OS, DSS, DFI, and PFI (*P* < 0.05). The R software packages “Survival” and “survminer” were used to plot the survival curve. The Kaplan–Meier survival analysis showed that the OS (*P* = 0.014; Figure 5(a)), DSS (*P* = 0.013; Figure 5(b)), DFI (*P* = 0.023; Figure 5(c)), and PFI (*P* = 0.001; Figure 5(d)) of cervical cancer patients with high PDIA3 expression were significantly lower than those with low expression, and patients with low PDIA3 expression had a longer survival time, suggesting that high PDIA3 expression was associated with a poor prognosis. These results indicated that the high expression of PDIA3 was correlated with the four survival indexes of OS, DSS, DFI, and PFI in patients with cervical cancer, and PDIA3 could be used as one of the indicators of poor prognosis in patients with cervical cancer.

### 3.6. Analysis of Correlation between PDIA3 Expression and DNA Methylation

**Figure 6:**
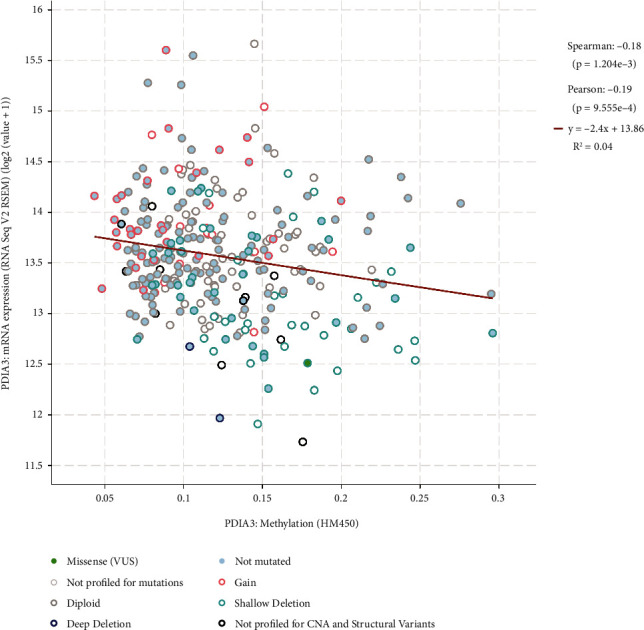
Correlation between PDIA3 expression and DNA methylation.

The changes in the genome level of PDIA3 were analyzed by cBioPortal database. The results showed that in the two selected groups of CESC studies, including 607 cervical cancer samples, the PDIA3 expression was negatively correlated with methylation level (Figure 6), and the difference was statistically significant (Spearman *r* = −0.18, *P* = 1.204 × 10^3^; Pearson *r* = −0.19, *P* = 9.555 × 10^−4^). This indicated that the high expression of PDIA3 in cervical cancer might be regulated by methylation modification.

### 3.7. Relationship between Expression Level of PDIA3 and Tumor Immune Cell Infiltration Level

**Figure 7:**
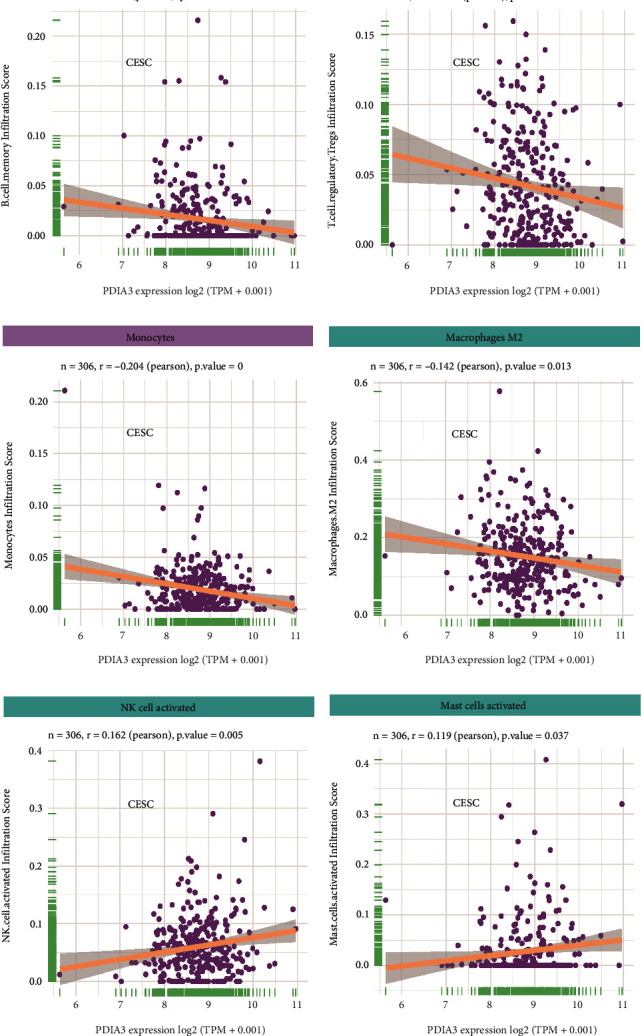
(a) Relationship between the PDIA3 expression and B cell memory in cervical cancer. (b) Relationship between the PDIA3 expression and T cell regulatory in cervical cancer. (c) Relationship between the PDIA3 expression and monocytes in cervical cancer. (d) Relationship between the PDIA3 expression and macrophages M2 in cervical cancer. (e) Relationship between the PDIA3 expression and NK cell activated in cervical cancer. (f) Relationship between the PDIA3 expression and mast cells activated in cervical cancer.

The R software packages “ggplot2”, “ggpubr,” and “ggExtra” were used to evaluate the correlation between the expression level of PDIA3 and 26 immune cell infiltration levels in cervical cancer. The results showed that the levels of PDIA3 expression in cervical cancer were negatively correlated with those of B cell memory (*r* = −0.132, *P* = 0.021; Figure 7(a)), T cell regulatory (*r* = −0.127, *P* = 0.026; Figure 7(b)), monocytes (*r* = −0.204, *P* = 0.; Figure 7(c)), and macrophages M2 (*r* = −0.142, *P* = 0.013; Figure 7(d)), but positively correlated with the levels of NK cell activated (*r* = 0.162, *P* = 0.005; Figure 7(e)) and mast cells activated (*r* = 0.119, *P* = 0.037; Figure 7(f)), both with statistically significant differences. Among them, the relationship with monocytes is the closest, with the most significant difference. These results suggest that PDIA3 may modulate cervical cancer occurrence and progression by affecting the six most critical types of immune cells: B cell memory, T cell regulatory, monocytes, macrophages M2, NK cell activated, and mast cells activated.

### 3.8. PPI Network Construction and Related Protein Interaction Analysis of PDIA3

**Figure 8:**
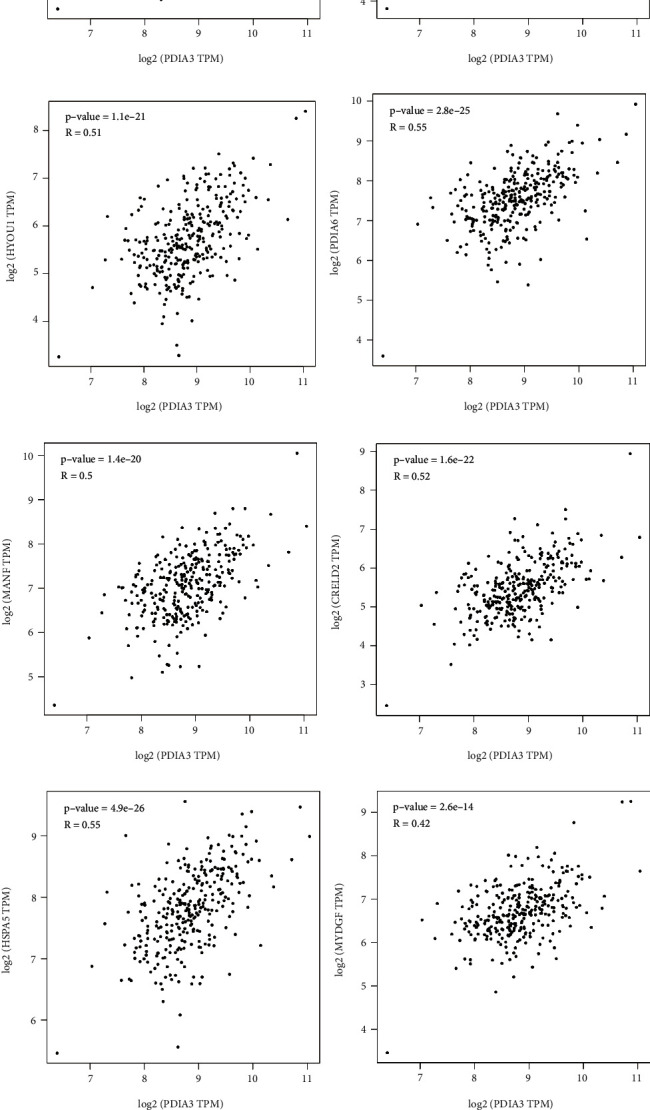
(a) Construction of PPI protein interaction network of PDIA3 gene. (b)–(k) Correlation between related genes and PDIA3 expression [(b): PPIB. (c): HSP90B1. (d): P4HB. (e): PDIA4. (f): HYOU1. (g): PDIA6. (h): MANF. (i): CRELD2. (j): HSPA5. (k): MYDGF (C19ORF10)]. (l) The expression of 10 target genes in each tumor.

The PPI network of PDIA3 constructed by STRING has 11 nodes, 42 sides, an average node degree of 7.64, and a PPI enrichment *P* = 6.41 × 10^−10^. The PPI-rich interacting proteins include HSP90B1, ERP27, HSPA5, CALR, STAT3, HLA-A, B2M, CANX, TAPBP, and TAP1 (Figure 8(a)). A total of 10 targeted binding proteins of PDIA3 and 10 genes related to PDIA3 expression were screened out for a series of correlation analysis of the molecular mechanism of PDIA3 in tumors. The PDIA3 expression levels were positively correlated with PPIB (*r* = 0.73; Figure 8(b)), HSP90B1 (*r* = 0.62; Figure 8(c)), P4HB (*r* = 0.49; Figure 8(d)), PDIA4 (*r* = 0.48; Figure 8(e)), HYOU1 (*r* = 0.51; Figure 8(f)), PDIA6 (*r* = 0.55; Figure 8(g)), MANF (*r* = 0.50; Figure 8(h)), CRELD2 (*r* = 0.52; Figure 8(i)), HSPA5 (*r* = 0.55; Figure 8(j)), and MYDGF (C19ORF10) (*r* = 0.42; Figure 8(k); *P* < 0.001). The corresponding heat map showed a positive correlation between PDIA3 and the above 10 genes in most cancers (Figure 8(l)).

### 3.9. Differential Co-Expressed Gene Screening and Functional Enrichment Analysis of PDIA3 in Cervical Cancer

**Figure 9:**
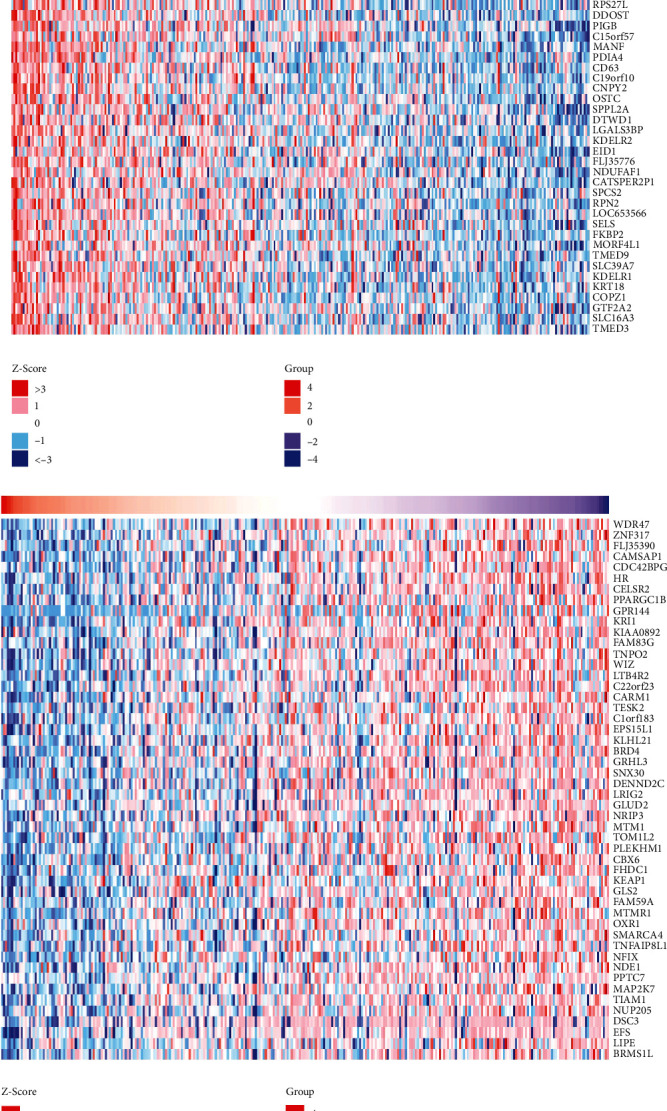
(a) PDIA3 differentially co-expressed top 50 positive related genes in cervical cancer. (b) PDIA3 differentially co-expressed top 50 negative related genes in cervical cancer.

**Figure 10:**
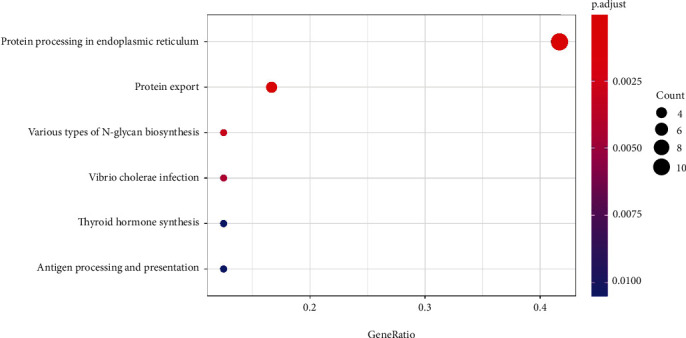
KEGG bubble plot of PDIA3 differentially co-expressed top 50 genes.

Based on the LinkedOmics database, the differentially co-expressed genes of PDIA3 in cervical cancer were analyzed and the Top 50 genes with significant difference (*P* < 0.05), and the closest correlation was screened out to draw the heat map, which showed that the genes significantly positively correlated with PDIA3 expression in cervical cancer included PDIA3P, PPIB, HSP90B1, SERF2, CALR, and HSPA5 (Figure 9(a)), and the genes significantly negatively correlated with PDIA3 expression included WDR47, ZNF317, FLJ35390, CAMSAP1, CDC42BPG, HR, and others (Figure 9(b)). Among them, HSP90B1, CALR, and HSPA5 in the Top 50 differentially co-expressed genes significantly positively correlated with PDIA3 also participated in the protein–protein interaction network. The first 50 differentially co-expressed genes most closely related to PDIA3 co-expressed genes screened out in the DAVID database were subjected to GO functional enrichment analysis based on DAVID database. These associated genes are mainly enriched in protein fold, protein transport, endoplasmic reticulum stress (ERS) response, reverse vesicle-mediated transport, ER protein fold, Chaperone-mediated protein fold, cellular redox homeostasis, ER–Golgi vesicle-mediated transport, ATF6-mediated unfolded protein response, protein retention in the ER lumen, signal peptide processing, *N*-linked protein glycosylation, ER-targeted protein, co-translational protein targeting, and other biological processes (Table 3). The KEGG analysis was mainly enriched in ER protein processing, protein transport, biosynthesis of various types of *N*-glycans, synthesis of thyroid hormone, and antigen processing and presentation pathways (Figure 10), among which the ER protein processing pathway was the most significant.

**Figure 11:**
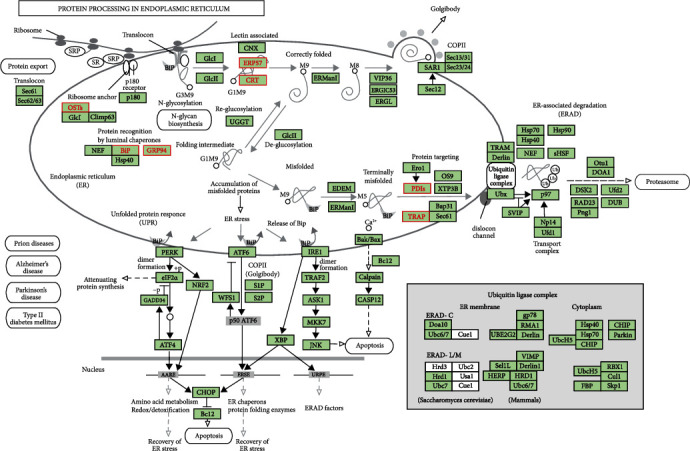
KEGG pathway of PDIA3 differentially co-expressed genes.

To further explore the roles of PDIA3 (ERP57) and related genes in ER protein processing pathway, the R software package “pathview” was used to plot the KEGG pathway map of the first 50 differential co-expressed genes screened that were the closest to PDIA3 co-expressed genes (Figure 11). The results showed that green were down-regulating genes, and red were up-regulating genes. The relevant positions of the corresponding genes in the pathway were marked with red markers, and seven related genes were related to ER protein processing. This indicates that PDIA3 (ERP57) in cervical cancer may regulate the occurrence and development of cervical cancer by regulating related molecules, affecting biosynthesis, and changing the related pathways, such as protein processing.

## 4. Discussion

According to GLOBOCAN 2020 cancer statistics worldwide, cervical cancer is the most common cancer in 23 countries and the leading cause of cancer deaths in 36 countries. In the correlation analysis between cervical cancer and HPV virus, HPV virus is found to be an important cause of 90% cervical cancer occurrence, affecting the treatment prognosis of patients [14–17]. Treatment options for cervical cancer patients continue to increase, with the emergence of tumor immunotherapy, targeted therapy, radiotherapy, chemotherapy, and surgical treatment. To date, the prognosis of patients has not significantly improved, and the mortality rate remains high [18]. Therefore, this study aims to screen out new prognostic markers of cervical cancer and reduce the mortality of cervical cancer patients, thereby providing reference for improving the prognosis and realizing individualized targeted therapy.

PDIA3 affects the occurrence and development of a variety of cancer tissues and regulates the proliferation of cancer cells, playing a vital role in the prognosis and treatment of malignant tumors [19]. The research by Zou et al. [20] suggested that patients with glioma with high PDIA3 expression had poor survival prognosis, and PDIA3 could be used as an independent prognostic biomarker of diffuse glioma, whose down-regulation might contribute to the survival of patients undergoing chemotherapy and radiotherapy. The research by Takata et al. and Liu et al. [21, 22] suggested that the high expression of PDIA3 presaged a poor prognosis for patients with liver cancer, and PDIA3 might be a key molecule in the developing new targeted therapies for liver cancer. The research by Liu et al. [23] also suggested that the high expression of PDIA3 in clear cell renal cell carcinoma was related to poor prognosis. Studies have shown that PDIA3 expression is related to the prognosis of ovarian cancer [24], gastric cancer [25], renal cancer, laryngeal cancer [26], breast cancer, cervical cancer, lung cancer [27], and prostate cancer. Studies in China and abroad have shown that there are differences in the expression of PDIA3/ERP57 in cervical cancer. The research by Hettinghouse et al. [28] showed that up-regulation or down-regulation of PDIA3 might be related to poor prognosis, depending on the affected tissues, and PDIA3 was an up-stream medium involved in key signaling pathways, such as tumorigenesis, invasion, cell proliferation, and apoptosis. The research by Liao et al. [29] suggested that the increased expression of PDIA3 in cervical cancer patients was related to poor prognosis. Rong et al. [30] proposed that the trend of high expression PDIA3 showed in cervical cancer tissues was positively correlated with the progression of cervical lesions. The Kaplan–Meier survival analysis showed that in cervical cancer patients, the OS, DSS, DFI, and PFI of patients with high expression of PDIA3 were significantly lower than those of patients with low expression, and the patients with low expression of PDIA3 had a long survival time, indicating that high expression of PDI3 was related to poor prognosis, which was consistent with the results of this study. However, Chung et al. [31] held that the lower the expression level of PDIA3/ERP57, the worse the prognosis. Therefore, combined detection should be selected as the relevant indicator for monitoring cervical lesions to predict the occurrence of cervical cancer and judge the prognosis. Therefore, PDIA3 is one of the reasonable candidate biomarkers for studying cancer prognosis and can be used as a target for cancer treatment [32].

Based on TIMER2.0 database, the expression of PDIA3 in 39 TCGA tumors was analyzed in this study. The results showed that PDIA3 was significantly overexpressed in 16 TCGA cancers (*P* < 0.05), and only lowly expressed in thyroid cancer tumor tissues, and it had strong immunoreactivity in cervical cancer tissues. Based on the GEPIA2 database, the expression of PDIA3 in 306 cervical cancer patients in the TCGA and GTEx databases was further verified. The results showed that the expression level of PDIA3 in cervical cancer tissues was significantly higher than that in normal tissues, which was consistent with the results of TIMER2.0 analysis. In addition, it is consistent with the results of immunohistochemistry. Further analysis of the relationship between PDIA3 and cervical cancer tumor staging based on the GEPIA2 database suggested that the expression of PDIA3 significantly increased with the progression of tumor staging. The experimental results showed that the expression level of PDIA3 in cervical cancer tissues was significantly higher than that in adjacent cancer tissues. The high or low expression of PDIA3 was significantly correlated with the clinicopathological classification of cervical cancer patients. The above analysis results indicate that PDIA3 is highly expressed in cervical cancer and can be used as a biomarker of poor prognosis of cervical cancer, and provide a new target for individualized diagnosis and treatment of cervical cancer.

The genomic level changes of PDIA3 were further analyzed through cBioPortal database to study the upstream mechanism of PDIA3 in cervical cancer. The results showed that the expression of PDIA3 was negatively correlated with methylation level, indicating that the high expression of PDIA3 in cervical cancer might be regulated by methylation modification. The relationship between PDIA3 expression level and immune-related cell infiltration level was analyzed for the downstream mechanism of PDIA3 in cervical cancer. The results showed that the expression level of PDIA3 was negatively correlated with the levels of infiltrating memory B cells, regulatory T cells, monocytes, and M2 macrophages, but positively correlated with the levels of NK cells and mast cells. Among them, the relationship with monocytes is the closest, with the most significant difference. These results indicated that PDIA3 might regulate the occurrence and development of cervical cancer by affecting the infiltration of the above six immune cells. Shibutani et al. and Giuliani et al. [33, 34] pointed out that the ratio of monocytes to lymphocytes could serve an indicator for clinical prediction of prognosis of cervical cancer patients, and monocytes were closely related to each tumor. Liu et al. and Veluchamy et al. [35, 36] believed that NK cells and regulatory T cells were independent factors affecting the occurrence and development of cervical cancer. Studies by Chen et al. [37] revealed that regulatory T cells had a high expression in cervical cancer tissues and were related to the malignancy and metastasis of tumor cells.

Based on the LinkedOmics database, the differentially co-expressed genes of PDIA3 in cervical cancer were analyzed, and the top5 0 genes with significant difference (*P* < 0.05) and the closest correlation were screened out to plot the heat map. The results showed that the genes, which significantly positively correlated with PDIA3 expression in cervical cancer, were PDIA3P, PPIB, HSP90B1, SERF2, CALR, and PDIA6, and the genes, which significantly negatively correlated with PDIA3 expression, were WDR47, ZNF317, FLJ35390, CAMSAP1, CDC42BPG, and HR. Based on the DAVID database, the top 50 differentially co-expressed genes most closely related to PDIA3 were subject to GO and KEGG functional enrichment analyses. The results showed that these associated genes were mainly enriched in ERS response, ATF6-mediated unfolded protein response, and other biological processes. Park and Ozcan [38] pointed out that ERS regulated the survival or apoptosis of tumor cells through IRE1*α*–XBP1, PERK–eIF2*α*, and ATF6 pathways, and ERS was activated in the occurrence and development of cervical cancer [35]. Yamamoto et al. and Hirose et al. [39, 40] believed that PDIA3 played the role of stress initiation factor in ERS and UPR, up-regulating the transcription of down-stream genes including PDIA3 to promote the initiation of UPR, which was related to the development, infiltration, and metastasis of esophageal squamous cell carcinoma, also closely related to the occurrence, development, metastasis, and prognosis of breast cancer, gastric cancer, liver cancer [41], CHOL, and prostate cancer.

In summary, this study has demonstrated that the expression of PDIA3 in cervical cancer is up-regulated, and the high expression of PDIA3 is related to tumor progression, which may be an independent prognostic biomarker of cervical cancer. At present, there are few studies on PDIA3 and cervical cancer, and the role and specific mechanism of PDIA3 in the occurrence and development of cervical cancer remain unclear. Therefore, in the future scientific research work, we will carry out in-depth discussion and research on PDIA3 with the view to provide the direction for the diagnosis, treatment, and prognosis of cervical cancer.

## Figures and Tables

**Table 1:** Expression of PDIA3 protein in cervical cancer tissues and adjacent cancer tissues [*n* (%)].

Tissues	*n*	PDIA3 protein	
		Low expression	High expression
Group data			
Cervical tissue	111	44 (39.6)	67 (60.4)
Pericarcinomatous tissue	24	16 (66.7)	8 (33.3)
*χ*^2^ value		4.7946	
*P*-value		0.02855	
Paired data			
Cervical cancer tissue	24	8 (33.3)	16 (66.7)
Pericarcinomatous tissue	24	16 (66.7)	8 (33.3)
*P*-value		0.04222	

**Table 2:** Relationship between PDIA3 protein expression and clinicopathological characteristics of cervical cancer [*n* (%)].

Clinicopathological parameters	*n*	PDIA3 expression [*n* (%)]		*P*-value
		Low	High	
Age (years)				
<50	76	26 (34.2)	50 (65.8)	0.1299
≥50	35	18 (51.4)	17 (48.6)	
Recurrence				
None	74	31 (41.9)	43 (58.1)	0.6311
Yes	37	13 (35.1)	24 (64.9)	
Pathological type				
Squamous carcinoma	85	36 (42.4)	49 (57.6)	0.0004
Adenocarcinoma	3	0 (0.0)	3 (100.0)	
Adenosquamous carcinoma	5	5 (100.0)	0 (0.0)	
Local squamous cell carcinoma	17	2 (11.8)	15 (88.2)	
Mixed adenocarcinoma	1	0 (100.0)	1 (0.0)	
T stage				
T1	62	22 (35.5)	40 (64.5)	0.4749
T2	29	12 (41.4)	17 (58.6)	
T3	19	10 (52.6)	9 (47.4)	
T4	1	0 (0.0)	1 (100.0)	
N stage				
N0	92	34 (37.0)	58 (63.0)	0.3105
N1	19	10 (52.6)	9 (47.4)	

**Table 3:** GO analysis of PDIA3 differentially co-expressed genes.

Category	Term	Count	*P*-value	FDR
GOTERM_BP_DIRECT	GO:0006457~protein folding	7	2.9 × 10^−6^	0.00029
	GO:0034975~protein folding in endoplasmic reticulum	4	3 × 10^−6^	0.00029
	GO:0034976~response to endoplasmic reticulum stress	5	2.4 × 10^−5^	0.00157
	GO:0006890~retrograde vesicle-mediated transport, Golgi to ER	5	3.4 × 10^−5^	0.00167
	GO:0061077~chaperone-mediated protein folding	4	7.9 × 10^−5^	0.00307
	GO:0036500~ATF6-mediated unfolded protein response	3	0.00018	0.00495
	GO:0006621~protein retention in ER lumen	3	0.00018	0.00495
	GO:0015031~protein transport	7	0.00024	0.0058
	GO:0045454~cell redox homeostasis	4	0.0007	0.0151
	GO:0006465~signal peptide processing	3	0.00145	0.02823
	GO:0018279~protein *N*-linked glycosylation via asparagine	3	0.00368	0.06531
	GO:0006888~ER to Golgi vesicle-mediated transport	4	0.00561	0.09115
	GO:0051208~sequestering of calcium ion	2	0.00902	0.13533
	GO:0045047~protein targeting to ER	2	0.02019	0.28119
	GO:0006613~cotranslational protein targeting to membrane	2	0.02241	0.29129
GOTERM_CC_DIRECT	GO:0005783~endoplasmic reticulum	20	2.4 × 10^−15^	2 × 10^−13^
	GO:0005789~endoplasmic reticulum membrane	17	1.9 × 10^−11^	5.7 × 10^−10^
	GO:0034663~endoplasmic reticulum chaperone complex	6	2 × 10^−11^	5.7 × 10^−10^
	GO:0042470~melanosome	8	2.5 × 10^−9^	5.2 × 10^−8^
	GO:0005788~endoplasmic reticulum lumen	9	9.3 × 10^−9^	1.5 × 10^−7^
	GO:0005793~endoplasmic reticulum-Golgi intermediate compartment	6	4.2 × 10^−7^	5.8 × 10^−6^
	GO:0030133~transport vesicle	5	6.1 × 10^−5^	0.00072
	GO:0070062~extracellular exosome	17	0.00021	0.00203
	GO:0008250~oligosaccharyltransferase complex	3	0.00022	0.00203
	GO:0005790~smooth endoplasmic reticulum	3	0.00155	0.01287
	GO:0005925~focal adhesion	6	0.00176	0.01328
	GO:0031012~extracellular matrix	5	0.0043	0.02978
	GO:0016020~membrane	12	0.00779	0.04971
	GO:0033116~endoplasmic reticulum~Golgi intermediate compartment membrane	3	0.00912	0.05404
	GO:0005623~cell	3	0.02127	0.11771
	GO:0030126~COPI vesicle coat	2	0.03105	0.16066
	GO:0009986~cell surface	5	0.03291	0.16066
	GO:0071682~endocytic vesicle lumen	2	0.03541	0.16328
	GO:0000139~Golgi membrane	5	0.04306	0.17892
	GO:0005794~Golgi apparatus	6	0.04311	0.17892
GOTERM_MF_DIRECT	GO:0003756~protein disulfide isomerase activity	4	1.6E-05	0.00127
	GO:0051082~unfolded protein binding	5	0.00011	0.0043
	GO:0004579~dolichyl-diphosphooligosaccharide-protein glycotransferase activity	3	0.00018	0.00475
	GO:0044822~poly(A) RNA binding	10	0.00072	0.01461
	GO:0005046~KDEL sequence binding	2	0.0045	0.07285
	GO:0016757~transferase activity, transferring glycosyl groups	3	0.00831	0.11212
	GO:0046923~ER retention sequence binding	2	0.01121	0.12967
	GO:0051087~chaperone binding	3	0.0143	0.14474
	GO:0051787~misfolded protein binding	2	0.02669	0.2402
	GO:0005515~protein binding	27	0.03026	0.24513
	GO:0050750~low-density lipoprotein particle receptor binding	2	0.03543	0.2609
	GO:0016853~isomerase activity	2	0.04625	0.31219

## Data Availability

The data analyzed in this study are available in the The Cancer Genome Atlas database (https://portal.gdc.cancer.gov/), TIMER2.0 database (https://www.proteinatlas.org/), Human Protein Atlas database (https://www.proteinatlas.org/), GEPIA2 database (http://gepia2.cancer-pku.cn/#analysis), GENCODE database (https://www.gencodegenes.org/), STRING database (http://string-db.org), GDC database (https://gdc.cancer.gov/about-data/publications//panimmune), cBioPortal database (http://www.cbioportal.org/), LinkedOmics database (http://www.Linkedomics.org), and DAVID database (https://david.ncifcrf.gov/).

## Additional Points

*Research Highlights*. (1) To study the expression and clinical significance of PDIA3 gene in patients with cervical cancer and its effect on prognosis. (2) There are few studies on PDIA3 and cervical cancer, and the role and specific mechanism of PDIA3 in the occurrence and development of cervical cancer remain unclear. (3) This study has demonstrated that the expression of PDIA3 in cervical cancer is up-regulated, and the high expression of PDIA3 is related to tumor progression, which may be an independent prognostic biomarker of cervical cancer.
